# Control of laser induced molecular fragmentation of *n*-propyl benzene using chirped femtosecond laser pulses

**DOI:** 10.1016/j.chemphys.2009.04.009

**Published:** 2009-06-12

**Authors:** Tapas Goswami, S.K. Karthick Kumar, Aveek Dutta, Debabrata Goswami

**Affiliations:** a Department of Chemistry, Indian Institute of Technology Kanpur, UP 208016, India; b Centre for Laser Technology, Indian Institute of Technology Kanpur, UP 208016, India

**Keywords:** Chirped femtosecond laser pulse, Fragmentation, Coherent control

## Abstract

We present the effect of chirping a femtosecond laser pulse on the fragmentation of *n*-propyl benzene. An enhancement of an order of magnitude for the relative yields of ${\text{C}}_{3}{\text{H}}_{3}^{+}$ and ${\text{C}}_{5}{\text{H}}_{5}^{+}$ in the case of negatively chirped pulses and ${\text{C}}_{6}{\text{H}}_{5}^{+}$ in the case of positively chirped pulses with respect to the transform-limited pulse indicates that in some fragmentation channel, coherence of the laser field plays an important role. For the relative yield of all other heavier fragment ions, resulting from the interaction of the intense laser field with the molecule, there is no such enhancement effect with the sign of chirp, within experimental errors. The importance of the laser phase is further reinforced through a direct comparison of the fragmentation results with the second harmonic of the chirped laser pulse with identical bandwidth.

## 1 Introduction

The control over the fragmentation and chemical reactions using shaped ultrafast laser pulses was demonstrated by different schemes of experiments by various groups of scientists [1–7]. All of their experiments are based on the closed loop approach using learning algorithms to control the laser field with feedback from the experimental signal. The resultant pulse shapes obtained by an adaptive approach were not always found to be a globally optimized solution [2,3,8,9]. Furthermore, the physical significance of such complex shaped pulses to the actual processes leading to discrimination among the fragmentation channels and their mechanisms are often too difficult to apprehend limiting the generalization of such feedback schemes. A continued effort, therefore, exists on the control of such fragmentation process from simpler pulse modulation concepts.

One of the easiest pulse modulation schemes is frequency chirping. Chirping essentially refers to the process of arranging the frequency components in a laser pulse with certain phase ordering. Linear ordering can be easily achieved by dispersing the ultrafast pulses through a pair of gratings. This ordering of frequency components results in the lengthening of an otherwise bandwidth limited ultrafast laser pulse. For positively chirped pulse leading edge of the pulse is red shifted and the trailing edge is blue shifted with respect to the central frequency of the pulse. Negative chirp corresponds to the opposite effect. The first experimental demonstration of control using simple linear chirped laser pulses was by Warren and co-workers in the early 1990s [10,11]. Subsequently, several experimental and theoretical developments have made linear chirped pulse control a very attractive field of research [12–17]. Control of fragmentation with simple linear chirped pulses as a control scheme has also become an active field of research in recent years [4,9,18–22].

It has been known [9,14,18,22] that the fragmentation processes in polyatomic molecules induced by an intense ultrafast laser field can sometimes exhibit sensitive dependence on the instantaneous phase characteristics of the laser field. Depending on the change in sign the chirped laser pulses, fragmentation could be either enhanced or suppressed [14,18,22]. Controlling the outcome of such laser induced molecular fragmentation with chirped femtosecond laser pulses has brought forth a number of experimental and theoretical effects in the recent years. However, efforts are continuing for a specific fragment channel enhancement, which is difficult since it also is a function of the molecular system under study [20,22–24]. Here we report the observation of a coherently enhanced fragmentation pathway of *n*-propyl benzene, which seems to have such specific fragmentation channel available. We found that for *n*-propyl benzene, the relative yield of ${\text{C}}_{3}{\text{H}}_{3}^{+}$ is extremely sensitive to the phase of the laser pulse as compared to any of the other possible channels. In fact, there is almost an order of magnitude enhancement in the yield of ${\text{C}}_{3}{\text{H}}_{3}^{+}$ when negatively chirped pulses are used, while there is no effect with the positive chirp. Moreover, the relative yield of all the other heavier fragment ions resulting from interaction of the strong field with the molecule is not sensitive to the sign of the chirp, within the noise level.

## 2 Experiment

**Fig. 1:**
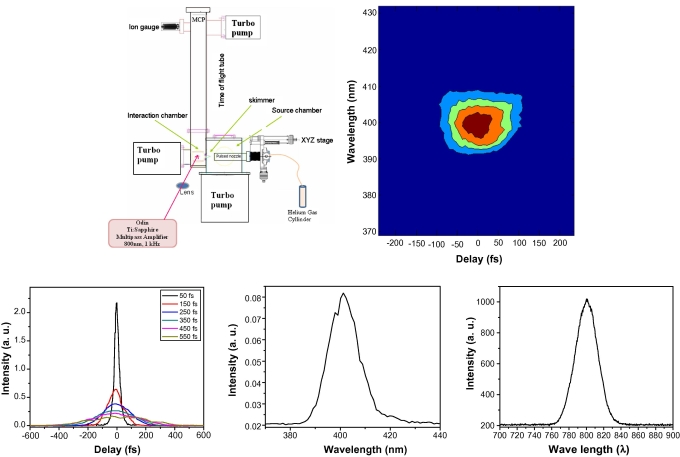
Schematic experimental setup. Inset (bottom center) shows the SHG-FROG of our typical near transform-limited amplified pulse collected using GRENOUILLE, and its corresponding spectral plot (bottom left). The various autocorrelation traces of the pulse for different chirps are shown in inset (bottom right).

The Laser system used in this experiment is a Ti:Sapphire multipass amplifier (Odin, Quantronix Inc.), which operates at 800 nm with 50 fs FWHM pulses at 1 kHz with energy of ∼1 mJ. It is seeded with a homebuilt Ti:Sapphire Oscillator (starting point: K&M Labs Inc. oscillator kit). The oscillator is pumped by a Nd:YVO_4_ (Verdi 5, Coherent Inc.), resulting in femtosecond pulses with a centre wavelength of 800 nm and a spectral bandwidth of 50 nm FWHM at 94 MHz repetition rate and an average power of 400 mW. The oscillator output is stretched and then fed into the Ti:Sapphire multipass amplifier which is pumped by the second harmonic of Nd:YAG laser operating at a repetition rate of 1 kHz (Corona, Coherent Inc.). The production of chirped ultrafast laser pulses is straightforward [18,25]: it is built-in our suitably modified compressor for the amplified laser system. As we increase the spacing between the compressor gratings relative to the optimum position for minimum pulse duration of 50 fs, we generate a negatively chirped pulse. Conversely, we obtain the positively chirped pulse by decreasing the inter-grating distance. Pulse durations were measured using a home-made intensity auto-correlation for the transform-limited pulse, as well as for the various negatively chirped and positively chirped pulses (Fig. 1 , inset bottom right). The pulses were further characterized by second harmonic frequency resolved optical gating (SHG-FROG) technique. Fig. 1 (inset bottom centre) shows a typical SHG-FROG trace of our near transform-limited pulse that was collected using GRENOUILLE (Swamp Optics Inc.). In Fig. 1 (inset bottom left), we show the SHG spectrum of the of the transform-limited pulses after frequency doubling with 50 μm type-1 BBO crystals as well as the spectrum of the transform-limited pulse collected with a HR-2000 spectrometer (Ocean Optics Inc.).

The laser pulses are then focused with a lens (focal length = 50 cm) on a supersonically expanded molecular beam of *n*-propyl benzene at the centre of a time of flight chamber. The polarization of the laser was horizontal as it enters the mass spectrometer and is perpendicular to ion collection optics. The design of our molecular beam setup (Fig. 1) consists of three chambers with differential pumping by turbo pumps. The three chambers were designed with differential pumping to attain a supersonic molecular beam region, a laser interaction region and a mass detection region, respectively. The source chamber or the supersonic molecular beam region is pumped by 2000 ls^−1^ turbo molecular pump (Varian Turbo-V2K-G). The ionization chamber (or the buffer chamber), where the laser interaction occurs, is pumped by a 500 l s^−1^ turbo pump (Varian Turbo-V551 Navigator). Finally, the mass detection region is pumped by a 300 l s^−1^ turbo pump (Varian Turbo-V301 Navigator). An engineering drawing of the system was prepared using AUTOCAD software. The vacuum chambers were fabricated based on our design and requirements for a complete oil-free system using SS-304 stainless steel and conflate connections. The pressure in the ionization chamber during the experiment was kept at 10^−7^ torr (base pressure of 5 × 10^−9^ torr). A pulsed valve (Series 9, General Valve) of 0.5 mm diameter operating at 10 Hz repetition rate and a skimmer of 1 mm diameter were used to introduce the supersonic molecular beam into the laser interaction region, where it is exposed to the intense laser field (1 kHz amplified Ti:Sapphire laser pulses as detailed earlier). The pulse duration of the molecular beam is kept at 100 μs. The molecular beam and laser pulses are synchronized by a delay generator. The *n*-propyl benzene sample at room temperature was used without further purification (98%; Sigma–Aldrich) and was seeded in Helium at 2 atm backing pressure. The mass spectra were recorded with a Wiley-McLaren type linear time of flight mass spectrometer. The ions are detected using an 18 mm dual micro-channel plate (MCP) detector coupled to a 1 GHz digital oscilloscope (Lecroy 6100A). The mass resolution of *n*-propyl benzene cation was calculated and was found to be *t*/2Δ*t* ∼ 1100. Mass spectra were typically averaged over 500 laser shots. The mass spectra from our particular beam chamber constructed with dry-scroll pumps and turbo-molecular pump as described above has the advantage that it does not contain any extraneous water and hydrocarbon peaks and thus has better sensitivity for organic samples as reported here.

## 3 Results and discussion

Study of aromatic hydrocarbons [26] has indicated different fragmentation channels. A systematic study of aromatic compounds with increasing chain-length of substituent alkyl groups has indicated that the fragmentation process is enhanced as the chain-length of the alkyl substituent on the benzene ring increases [26]. We have chosen to apply chirped pulse fragmentation control on certain members of these systematically studied aromatic compounds. In general, as reported previously for benzene and toluene, *p*-nitro toluene [22], we also find that chirping favors the formation of smaller fragments as compared to the heavier ones. However, *n*-propyl benzene has the unique property of enhancing a particular fragmentation channel under the effect of chirp.

The phase (*φ*(*ω*)) of the laser pulse which is centered at *ω*_0_, can be expanded around *ω*_0_ to second order in *ω*:

(1) $$\varphi (\omega )≃\varphi (\omega_{0})+\frac{1}{1!}{\left(\frac{\partial \varphi }{\partial \omega }\right|}_{\omega =\omega_{0}}(\omega -\omega_{0})+\frac{1}{2!}{\left(\frac{\partial^{2}\varphi }{\partial \omega^{2}}\right|}_{\omega =\omega_{0}}{(\omega -\omega_{0})}^{2}\text{.}$$

Here, the second order term is responsible for group velocity dispersion. In fact, $\beta ={\left(\frac{\partial^{2}\varphi }{\partial \omega^{2}}\right|}_{\omega =\omega_{0}}$ is linear chirp coefficient (chirp parameter in the frequency domain) introduced by the compressor and is defined as second derivative of the spectral phase at the center frequency. The linear chirp coefficient (*β*) can be calculated using the equation [27–29]: $\tau^{2}=\tau_{0}^{2}+{\left[\frac{4\beta \ln2}{\tau_{0}}\right]}^{2}$, where *τ* is the pulse duration of the chirped laser pulse and *τ*_0_ is the chirp-free pulse duration of the transform-limited pulse in FWHM. The experimental error in the chirp value calculated from the equation mentioned above is about ±9%.

**Fig. 2:**
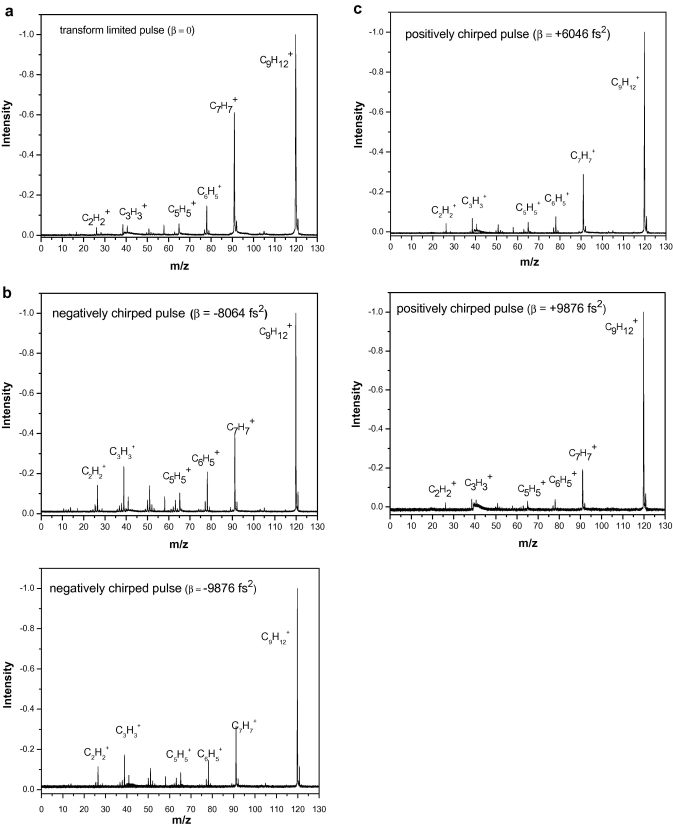
Effect of chirping the pulse on mass spectra of *n*-propyl benzene. (a) Mass spectra of *n*-propyl benzene when the laser pulse is transform-limited (*β* = 0). (b) Mass spectra of *n*-propyl benzene with negatively chirped pulse (*β* = −8064 fs^2^ and *β* = −9876 fs^2^). (c) Mass spectra of *n*-propyl benzene with positively chirped pulse (*β* = +6046 fs^2^ and *β* = +9876 fs^2^).

**Fig. 3:**
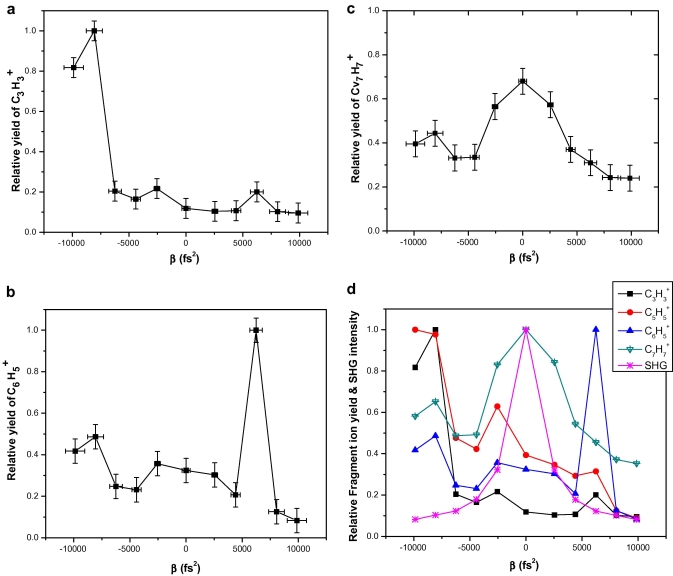
Effect of chirping the pulse on relative yield of (a) ${\text{C}}_{3}{\text{H}}_{3}^{+}$, (b) ${\text{C}}_{6}{\text{H}}_{5}^{+}$ and (c) ${\text{C}}_{7}{\text{H}}_{7}^{+}$. (d) Effect of chirping the laser pulse on the relative yield of different fragment ions shown in comparison to the integrated SHG intensity at the respective chirps.

We record the TOF mass spectra (Fig. 2 ) of *n*-propyl benzene using linearly chirped and unchirped ultrafast laser pulses with constant average energy of ∼200 mW. Next, we compared the corresponding peaks in mass spectra by calculating their respective integrals under the peaks and normalizing them with respect to the molecular ion. These results also conform to the case when we just compare the simple heights of the individual peaks. When the linear chirp of the laser pulse is negative, the relative yields of the smaller fragment ion, such as, ${\text{C}}_{3}{\text{H}}_{3}^{+}$ (mass to charge ratio, i.e., *m*/*z* = 39) is largely different from those obtained using transform-limited pulses or from the positively chirped pulses, as reflected in Fig. 3 a. The relative yield of ${\text{C}}_{3}{\text{H}}_{3}^{+}$ reaches maximum when the linear chirp coefficient (*β*, calculated by using the equation as mention earlier) is −8064 fs^2^ and pulse duration is of 450 fs. We would like to point out that the fragment ion ${\text{C}}_{6}{\text{H}}_{5}^{+}$ (*m*/*z* = 77) yield is more when the chirp is positive (*β* = +6246 fs^2^), and this can be attributed to a different fragmentation pathway [18] (Fig. 3b). However, the observation of enhancement for only one chirp sign implies that the observed enhancements are not due to the pulse width effects, they rather depend on the magnitude and sign of the chirp [2,4]. Hence coherence of the laser field plays an important role in this photofragmentaion process. It is also seen that relative yields of the heavier fragments like ${\text{C}}_{7}{\text{H}}_{7}^{+}$ (*m*/*z* = 91) is not affected by the sign of the chirp. The relative yield of ${\text{C}}_{7}{\text{H}}_{7}^{+}$ decreased in both the directions of the chirp and is at its maximum when the pulse is transform-limited (Fig. 3c), indicating that the fragment yield only depends on the laser peak intensity as dictated by its pulse width. We have also seen that the integrated SHG intensity at different chirp is symmetrically decaying around 0 fs^2^ (Fig. 3d), which confirms that there is nothing systematic in the laser pulse causing the enhancements in the fragmentation process.

Many experimental efforts have been made in recent years to control the fragmentation using chirped laser pulses. A general conclusion from several recent studies [9,14,18,22] is that the fragmentation process is preferred over the formation of molecular ion as the magnitude of chirp is increased. Our results also obey the same conclusion. Recently, Dantus and co-workers [22–24] also found out that change in the fragmentation with chirped laser pulses depends on the molecular structure. This is clearly evident in our results. Dantus et al. have shown that fragmentation of *p*-nitro toluene is enhanced when chirped laser pulses were used. The enhancement of the fragment ions of *p*-nitro toluene, such as, ${\text{C}}_{3}{\text{H}}_{3}^{+}$, ${\text{C}}_{5}{\text{H}}_{5}^{+}$ was independent of the sign of the chirp but some smaller fragment like ${\text{NO}}_{2}^{+}$ does depend on the sign of the chirp and it gets maximized when the chirp is negative.

**Fig. 4:**
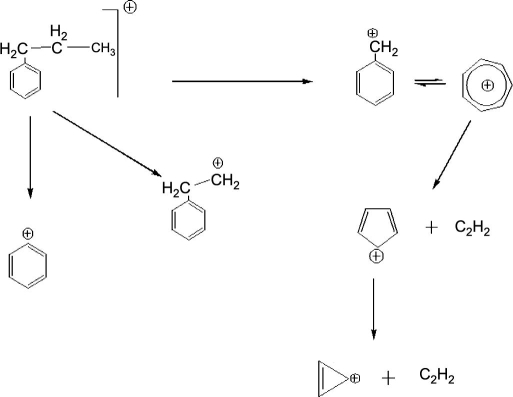
Fragmentation mechanism of *n*-propyl benzene.

As discussed by Lozovoy and Dantus [22], the mechanism of fragmentation of *p*-nitro toluene follows these steps: first, an ionization of the parent molecule occurs; next is the isomerization of *p*-nitro toluene ion, which is followed by the elimination of ${\text{NO}}_{2}^{+}$ ion leading to ${\text{C}}_{7}{\text{H}}_{7}^{+}$ tropylium ion; and this finally dissociates to form ${\text{C}}_{3}{\text{H}}_{3}^{+}$ ion. However, in case of *n*-propyl benzene, ${\text{C}}_{3}{\text{H}}_{3}^{+}$ ions are generated from ${\text{C}}_{7}{\text{H}}_{7}^{+}$ ions that form from the ionization of the parent molecule followed by the elimination of ${\text{C}}_{2}{\text{H}}_{5}^{+}$ ions. There is a large energy barrier (∼9 eV) that needs to be surmounted to achieve ionization of molecules like *p*-nitro toluene. The laser intensity needed for ionization causes excitation in a large number of intermediate rovibronic states some of which include dissociation continua. Therefore, the resulting coherent superposition undergoes very fast delocalization. This was one of the reasons why fragmentation does not depend on the parameter, time delay between sub-pulses, in the case of *p*-nitro toluene. In our case, for *n*-propyl benzene, the fragmentation mechanism (Fig. 4 ) is almost same, but it does not have such energy barrier, and also due to the longer alkyl chain-length, energy redistribution is relatively faster than the other alkyl derivative of benzene. Thus, fragmentation is more, which easily leads to dissociation into ${\text{C}}_{3}{\text{H}}_{3}^{+}$. We think this can be the reason why fragmentation pattern of *n*-propyl benzene shows the dependence on the sign of the chirp.

We should also consider the fact that the chirped pulse gives a linear delay between high and low frequencies within the bandwidth of the laser pulses. This temporal progression can be linked to pump–dump processes [13,30]. Varying the time delay between the high and low frequencies within the band width of the laser pulses, may coherently drive the vibrations that could be implicated in selective bond cleavage. Fragmentation pattern depend upon the molecular potential energy surface in the optical field and also on the efficiency with which energy from the optical field is coupled into the molecule. A red-blue sequence of photon interaction starts and ends at different points of PES than does a blue-red sequence, and thus there is ample reason to believe that the negative chirp will enhance the process of fragmentation as it results in a better overlap between the pump and dump pulses as compared to that of the transform-limited case.

## 4 Conclusion

We have demonstrated that the phase characteristics of the femtosecond laser pulse play a very important role in the laser induced fragmentation of polyatomic molecules like *n*-propyl benzene. The use of chirped pulse leads to sufficient differences in the fragmentation pattern of *n*-propyl benzene, so that it is possible to control a particular fragmentation channel with chirped pulses. Overall, as compared to the transform-limited pulse, negatively chirped pulses enhance the relative yield of ${\text{C}}_{3}{\text{H}}_{3}^{+}$, ${\text{C}}_{5}{\text{H}}_{5}^{+}$, while the relative yield of ${\text{C}}_{6}{\text{H}}_{5}^{+}$ increases in case of positively chirped pulse. In fact, for the ${\text{C}}_{3}{\text{H}}_{3}^{+}$ fragment, the enhancement is almost six times for the negatively chirped pulse (*β* = −8064 fs^2^) as compared to that of the transform-limited pulse.
