# Supplementary material for: Control of laser induced molecular fragmentation of n-propyl benzene using chirped femtosecond laser pulses
Source: Chem Phys. 2009 Jun 12;360(1-3):47–52. doi: 10.1016/j.chemphys.2009.04.009 (PMC2722903; doi:10.1016/j.chemphys.2009.04.009)
Supplement: Supplementary data [file mmc1.doc]

Supplementary Information

Supplementary Figure: Retrieved Spectra and Phase from the GRENOUILLE-FROG in Fig. 1 (inset center) of the manuscript
